# Topical Cycling and Systemic Eligibility in Patients With Psoriasis in Japan

**DOI:** 10.1111/1346-8138.70395

**Published:** 2026-07-24

**Authors:** Akimichi Morita, Shinichi Imafuku, Hideshi Torii, Lauren Peetluk, Siddharth Chaudhari, Hidehito Mizuma, Jing Liu

**Affiliations:** ^1^ Nagoya City University Nagoya Japan; ^2^ Fukuoka University Fukuoka Japan; ^3^ Tokyo Yamate Medical Center Tokyo Japan; ^4^ Amgen Inc Thousand Oaks California USA; ^5^ Amgen K.K. Tokyo Japan

**Keywords:** Japan, psoriasis, treatment switching

## Abstract

Topical therapy is the typical first‐line treatment for psoriasis, with the International Psoriasis Council recommending systemic therapy for patients with body surface area > 10%, high‐impact site involvement, or topical therapy failure. A recent Japanese Delphi Consensus Study proposed a definition for topical failure that considers time on therapy, failure to respond, flare on therapy, and other concepts. Using 2 real‐world data sources, this study aimed to describe topical cycling and evaluate systemic therapy eligibility among patients with psoriasis in Japan. With DeSC insurance claims data (January 1, 2017, to August 31, 2024), we evaluated topical therapy treatment patterns before systemic treatment initiation. With data from the Japanese subgroup of the UPLIFT survey, we applied recommendations from the Japan Delphi Study to estimate the proportion of patients receiving topical therapy who may be eligible for systemic treatment. Of 6361 patients with psoriasis from the DeSC database, at least 1 dispensing of a topical therapy (index event) and no systemic treatment at index, 9.5% initiated systemic therapy during follow‐up (mean time to initiation 712.5 days). Most patients began with strong/very strong corticosteroids (35.0%) or combinations of topical therapies (± vitamin D; 41.8%), with a shift toward predominantly combination regimens during follow‐up. Most patients received multiple topical therapy classes before initiating systemic therapy (mean unique classes: 3.8). Of 137 Japanese UPLIFT survey respondents with psoriasis with or without psoriatic arthritis using only topical therapy, 92.7% met at least 1 of the 7 Delphi consensus recommendations assessed, indicating potential eligibility for systemic treatment; 87.6% met more than 1 recommendation and 57.7% met 3 or more. Our findings suggest that many Japanese adults with psoriasis who may be eligible for systemic therapy may be undertreated and highlight the need to apply eligibility criteria for systemic treatment in clinical practice, which could improve long‐term patient outcomes.

## Graphical abstract

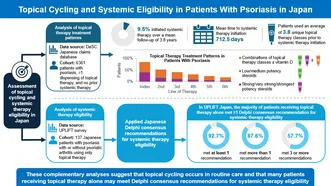

## 1 Introduction

Psoriasis (PsO) is a chronic, systemic, inflammatory disease [1]. In addition to skin lesions, approximately 30% of patients with PsO develop psoriatic arthritis (PsA) [2]. Furthermore, due to the systemic nature of the disease, PsO is associated with a substantial comorbidity burden [3]. For example, patients with PsO have a greater risk of obesity, diabetes, and cardiometabolic disease [4, 5, 6, 7]. Depression, anxiety, inflammatory bowel disease, and uveitis are also considered comorbidities of PsO [5, 8, 9].

Topical therapy is the typical first‐line treatment for PsO, with the International Psoriasis Council (IPC) recommending systemic (oral or injectable) therapy for patients with affected body surface area (BSA) > 10%, involvement of high‐impact sites, or topical therapy failure [10, 11]. Topical failure was undefined until a recent Japanese Delphi Consensus Study proposed a definition which, among other concepts, considers time on therapy, failure to respond, and flare on therapy [12]. Furthermore, consensus statements from this Delphi study recommend reviewing disease severity, disease control, and treatment satisfaction in patients who have used 2 or more topical therapies within 4 to 8 weeks. The IPC also recently issued guidance defining topical failure as inability to achieve clear/nearly clear skin (BSA ≤ 1% or Physician's Global Assessment [PGA] 0 or 1) after 2 consecutive 4‐week topical therapy courses [13].

The Understanding Psoriatic Disease Leveraging Insights for Treatment (UPLIFT) survey was a cross‐sectional online survey conducted in Europe, North America, and Japan from March to June 2020 [14]. The study aimed to better understand PsO disease burden and treatment expectations from the perspectives of both dermatologists and patients reporting a diagnosis of PsO and/or PsA. A subgroup analysis of Japanese patients with PsO from UPLIFT demonstrated that BSA underestimated patient perception of disease severity, resulting in undertreatment [15]. In the Japanese subgroup of UPLIFT, 25.0% of patients with BSA > 10% were treated with topical therapy only [15]. Furthermore, in a Japanese epidemiological survey, topical therapy was the most commonly used treatment, reported by 68.9% of patients with PsO [16]. By contrast, oral therapies were used by 26.6% of patients and biologics by 18.6%. Persistent cycling between topical therapies may reflect unresolved disease burden and delayed treatment optimization, and delayed systemic treatment has been associated with worse long‐term prognosis [17, 18, 19]. Despite many patients receiving only topical treatments, there is a lack of studies on topical therapy treatment patterns in Japan as well as a lack of data quantifying the proportion of systemic eligible patients with PsO who remain on topical therapy in Japan.

Using 2 complementary real‐world data sources, this study aims to characterize the unmet need for systemic therapy among patients with PsO in Japan. Specifically, our study objectives were to (1) evaluate real‐world topical therapy treatment patterns before initiation of systemic treatment in Japan using claims data, including sequential switching among topical therapies (topical “cycling”); and (2) apply the Japanese Delphi consensus recommendations for PsO to the Japan UPLIFT subgroup and assess the proportion of patients eligible for systemic therapy. The claims data analysis provides a population‐based evaluation of topical treatment patterns, enabling description of topical cycling and longitudinal treatment pathways in routine care. In parallel, survey data from UPLIFT allow direct assessment of clinically meaningful indicators of topical therapy failure and systemic eligibility by mapping patient‐reported data to Delphi consensus recommendations. Together, these data sources provide breadth and clinical interpretability to support a comprehensive assessment of topical cycling and systemic eligibility among patients with PsO in Japan.

## 2 Methods

### 2.1 Study Design

#### 2.1.1 DeSC Claims Analysis

The primary analysis was a retrospective analysis of patient‐level data (January 1, 2017, to August 31, 2024) from the DeSC claims database. Patients were followed from index date (first observed dispensing of a topical therapy) until PsO treatment discontinuation, disenrollment, or death (Figure S1A). The DeSC database is a payer‐based database of claims data provided by 3 prominent insurance systems in Japan, including Employee Health Insurance for salaried employees, National Health Insurance for self‐employed or retired people, and Latter‐Stage Elderly Healthcare System for people aged 75 years and older. The data include de‐identified eligibility, diagnosis, medication, and procedure data for enrolled individuals.

#### 2.1.2 UPLIFT‐Delphi Analysis

A secondary analysis used the Japanese subgroup of the UPLIFT survey (*N* = 391) [15] to assess the percentage of patients meeting each of the Japanese Delphi consensus recommendations for systemic treatment eligibility (Figure S1B) [12]. UPLIFT was a multinational, cross‐sectional, online survey conducted from March to June 2020 [14], with the study design and eligibility criteria from the Japanese subgroup published previously [15].

### 2.2 Eligibility Criteria

#### 2.2.1 DeSC Claims Analysis

From the DeSC database, we included adults ≥ 18 years of age with at least 1 recorded dispensing of a topical therapy from January 1, 2017, through August 31, 2024; no systemic treatment prior to or on the index date; a diagnosis of plaque PsO, defined as ≥ 1 inpatient or ≥ 2 outpatient claims with a diagnosis of PsO between 30 and 365 days apart on or before the index date; and at least 180 days of claims enrollment before the index date (baseline period). PsO diagnoses were assessed in inpatient and outpatient claims using all available baseline data, and were identified via International Classification of Disease, 9th Revision, Clinical Modification (ICD‐9‐CM) codes, ICD‐10‐CM codes, or native disease codes for Japan.

#### 2.2.2 UPLIFT‐Delphi Analysis

Adults with a self‐reported healthcare provider diagnosis of PsO and/or PsA were included in the UPLIFT study [14]. The secondary analysis included Japanese patients from UPLIFT with a diagnosis of PsO or PsO and PsA who were receiving only topical therapy at the time of the survey.

### 2.3 Assessments

#### 2.3.1 DeSC Claims Analysis

Among patients with PsO receiving topical therapy in clinical practice, we (1) describe treatment patterns, including topical and systemic therapies, and indicators for potential eligibility for systemic treatment based on the Japanese Delphi Consensus Study and IPC guidance on defining topical failure; (2) describe baseline patient and clinical characteristics; (3) assess incidence of patients initiating systemic therapy over all available follow‐up, and (4) compare treatment patterns and patient and clinical characteristics in patients who do and do not initiate systemic therapy during the follow‐up period.

Initiation of systemic therapy was considered an indicator for insufficient response to topical treatment, although it should be noted that treatment escalation can also reflect physician/patient preference, comorbidities/contraindications, or severity factors not captured in claims. Duration of PsO was defined as the time between first observed PsO diagnosis in the claims database and the index date. Comorbidities were defined using ICD‐10‐CM codes; ≥ 1 inpatient claim or ≥ 2 outpatient claims were required for a comorbidity to be considered present at baseline. Charlson Comorbidity Index (CCI) was evaluated at index date based on a validated algorithm [20]. Psoriasis Area and Severity Index > 3 or PGA ≥ 2 after 8 weeks of therapy with 2 or more topical medications, poor patient satisfaction or inadequate symptom control after 4 weeks with 2 or more topical medications constitute topical failure per the Japanese Delphi consensus recommendations [12]. To quantify these key milestones for assessing response to topical therapy, we also evaluate the average number of topical medications used within 8 weeks, the proportion of individuals who used more than 2 topical medications within 8 weeks, and the average time to initiation of a third topical medication.

Topical therapies were grouped into the following medication classes: low/medium potency topical steroid, strong/very strong potency topical steroid, strongest potency topical steroid, vitamin D analogues, and fixed dose combination of vitamin D and topical steroids. The first line of topical therapy was defined by topical medication class or classes dispensed on the same day. This line of therapy continued until the earliest of (1) dispensing of a new medication class or (2) 90 days after the most recent dispensing unless an additional dispensing within the same medication class occurred before that time. If, after the first line of therapy, an individual was dispensed a new medication class, a new line of therapy was initiated. If the overlap between the new and prior medication classes was > 30 days, the new line of therapy was considered combination therapy including both the prior and new classes; if the overlap was ≤ 30 days, the prior medication class was assumed to be discontinued, and the new line of therapy consisted only of the new medication class. When subsequent dispensings occurred within the same medication class without interruption, the line of therapy was extended and ended 90 days after the last uninterrupted dispensing for that class.

#### 2.3.2 UPLIFT‐Delphi Analysis

UPLIFT survey data approximating the Japanese Delphi consensus recommendations were considered proxies for those recommendations (Figure S2). Using these proxies, we estimated the size of the systemic eligible population in the Japanese subgroup of UPLIFT. Based on these consensus recommendations, we considered the following factors to warrant systemic treatment in patients with BSA < 10%: involvement of special areas; failure of topical therapy (based on inadequate symptom/skin control, poor satisfaction, and disease flare); PsO‐induced psychological distress; negatively impacted social life; presence of PsA; and risk of developing PsA (including nail psoriasis and family history [12]). We then used the following data proxies from UPLIFT to identify systemic eligible patients meeting the Delphi consensus recommendations: patient‐reported PsO symptoms and symptom burden, patient‐perceived PsO disease severity, patient‐reported treatment satisfaction, patient‐reported PsO symptom location, potential PsA based on the Psoriasis Epidemiology Screening Tool, patient‐perceived quality of life based on Dermatology Quality of Life Index (DLQI), and depression score based on Patient Health Questionnaire‐2 (PHQ‐2). A list of the Japanese Delphi consensus recommendations and the data proxies from UPLIFT that were used is shown in Figure S2.

### 2.4 Statistical Analysis

All analyses were descriptive; no formal hypotheses were tested. Sankey diagrams were used to assess treatment patterns during follow‐up.

## 3 Results

### 3.1 DeSC Claims Analysis

A total of 6361 patients from the DeSC database met the inclusion criteria and were included in the primary analysis (Figure S3).

**FIGURE 1:**
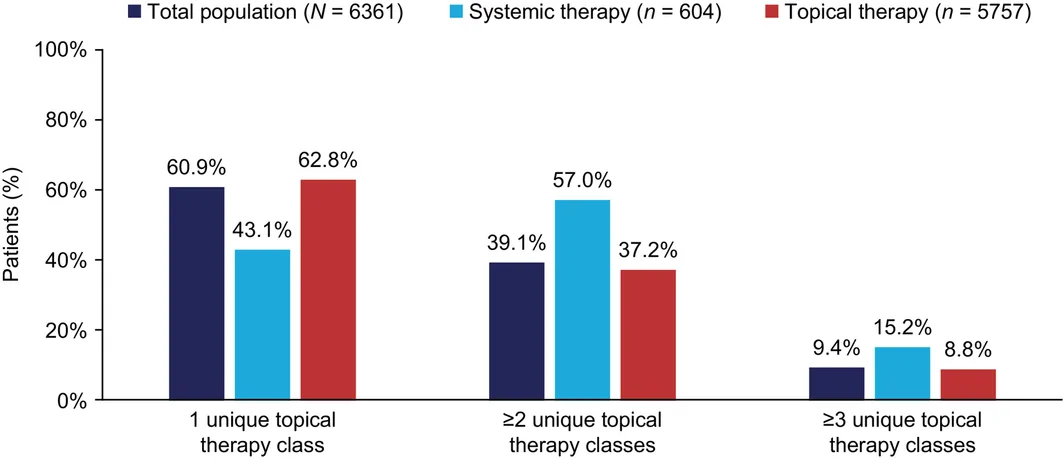
Number of lines of topical treatment at index. Categories are not mutually exclusive (i.e., patients included in the ≥ 3 topical therapy classes group are also included in the ≥ 2 topical therapy classes group).

Overall, mean age was 66.8 years, 58.3% were male, mean duration of PsO at index was 14.5 months, and 604 (9.5%) patients initiated systemic therapy during follow‐up (Table 1). Most (81.3%) patients were initially treated in an outpatient setting. The most common (≥ 5%) comorbidities were hypertension (10.4%), malignancies (6.5%), cerebrovascular disease (5.3%), diabetes (5.1%), and dyslipidemia (5.0%); 28.0% of patients had a CCI ≥ 1, including 24.0% of those who initiated systemic therapy and 28.4% of those who were treated with topical therapy alone. Patients who initiated systemic therapy during follow‐up had more complex topical use at index, with 57.0% of those patients receiving ≥ 2 unique topical therapy classes at index versus 37.2% of patients who were treated with topical therapy alone (Figure 1). Mean (standard deviation [SD]) duration of follow‐up was 3.8 (2.3) years.

**TABLE 1:** Patient characteristics (DeSC analysis of patients with PsO receiving topical therapy).

	Total population *N* = 6361	Systemic therapy initiators *n* = 604	Topical therapy alone *n* = 5757
Age at index, years, mean (SD)	66.8 (16.5)	60.7 (15.8)	67.4 (16.4)
Male, *n* (%)	3707 (58.3)	386 (63.9)	3321 (57.7)
Female, *n* (%)	2654 (41.7)	218 (36.1)	2436 (42.3)
BMI, kg/m^2^, mean (SD)	23.8 (3.9)	23.9 (3.9)	23.7 (3.9)
Duration of PsO, months, mean (SD)	14.5 (23.4)	14.1 (19.4)	14.6 (23.8)
Facility type of initial PsO treatment, *n* (%)			
Inpatient	1189 (18.7)	58 (9.6)	1131 (19.7)
Outpatient	5172 (81.3)	546 (90.4)	4626 (80.4)
CCI ≥ 1, *n* (%)	1778 (28.0)	145 (24.0)	1633 (28.4)
Comorbidities, *n* (%)			
Depression	129 (2.0)	7 (1.2)	122 (2.1)
Diabetes	323 (5.1)	20 (3.3)	303 (5.3)
Dyslipidemia	315 (5.0)	22 (3.6)	293 (5.1)
Hypertension	661 (10.4)	38 (6.3)	623 (10.8)
Cerebrovascular disease	336 (5.3)	16 (2.7)	320 (5.6)
Malignancies	411 (6.5)	13 (2.2)	398 (6.9)

Abbreviations: BMI, body mass index; CCI, Charlson Comorbidity Index; PsO, psoriasis; SD, standard deviation.

**FIGURE 2:**
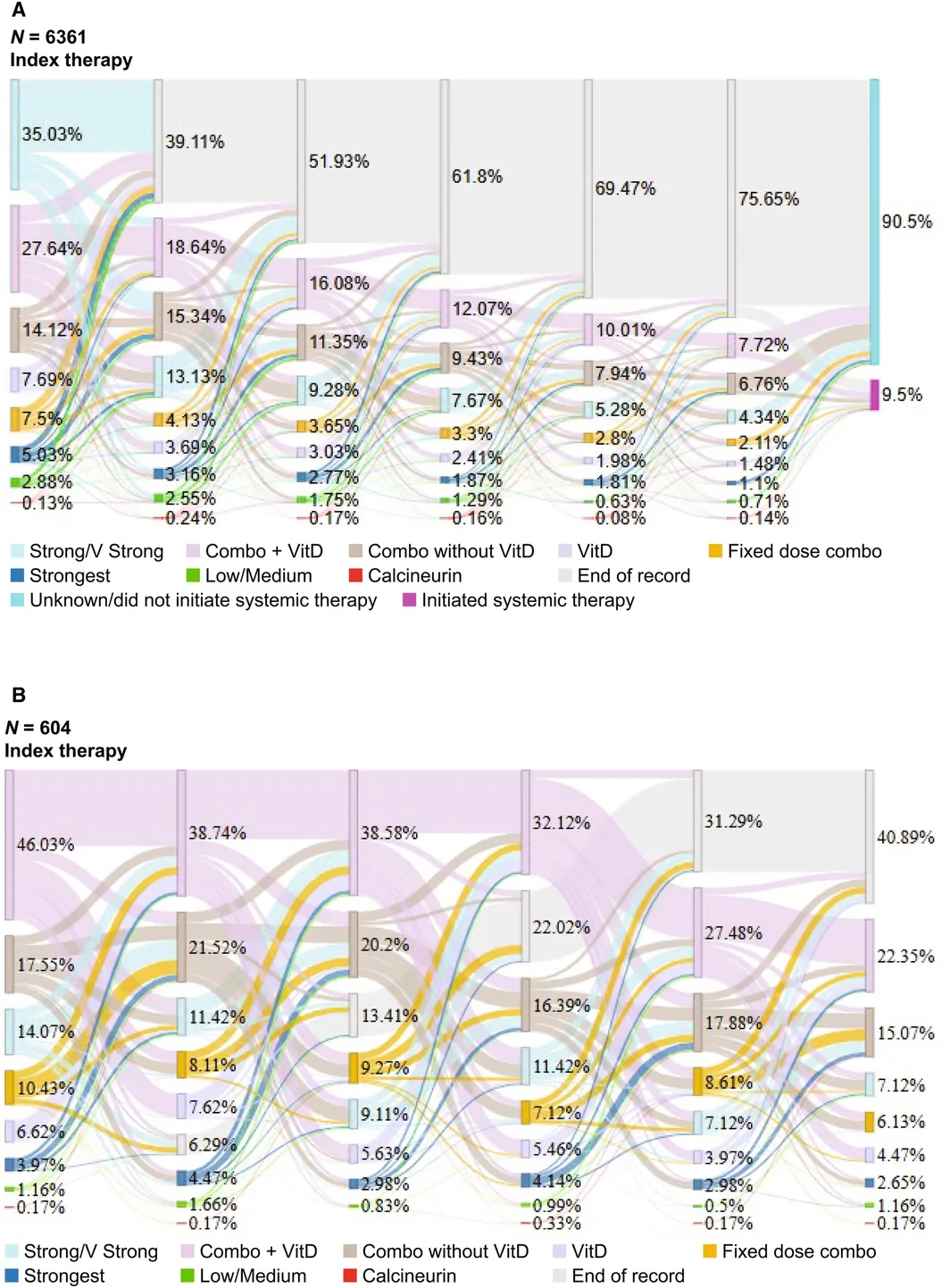
Progression of treatment over time. (A) Total population. (B) Patients who initiated systemic therapy. “Combo” refers to combinations of different topical therapies. “Fixed dose combo” refers to the fixed‐dose combination with vitamin D. “Low,” “Medium,” “Strong,” “V Strong,” and “Strongest” refer to topical steroid potency [21, 22]. “End of record” indicates patients who disenrolled, discontinued topical therapy, or died during follow‐up. V, very; VitD, vitamin D.

Overall, most patients began with either strong/very strong topical corticosteroids (35.0%) or combinations of topical therapies (± vitamin D; 41.8%), with a gradual shift toward predominantly combination regimens during follow‐up (Figure 2A). Notably, 75.7% of all patients either disenrolled, discontinued topical therapy, or died during follow‐up (“end of record”). Topical therapy changes preceding the switch to systemic therapy are shown in Figure 2B. Mean (SD) time to first systemic treatment initiation was 712.5 (622.8) days (approximately 2 years), with 76.2% of patients initiating systemic therapy > 180 days after PsO diagnosis. Most (93.9%) patients received multiple topical therapy classes before initiating systemic therapy (Table 2); 20.2% of patients received 5 cycles of topical therapy and 11.4% received ≥ 6 cycles (mean [SD] unique classes: 3.8 [1.4]).

**TABLE 2:** Follow‐up therapy and indicators for systemic therapy eligibility.

	Total population *N* = 6361	Systemic therapy initiators *n* = 604	Topical therapy alone *n* = 5757
Number of unique topical therapy classes used during follow‐up, *n* (%)			
0	850 (13.4)	10 (1.7)	840 (14.6)
1	1287 (20.2)	27 (4.5)	1260 (21.9)
2	1432 (22.5)	76 (12.6)	1356 (23.6)
3	1220 (19.2)	150 (24.8)	1070 (18.6)
4	890 (14.0)	150 (24.8)	740 (12.9)
5	442 (7.0)	122 (20.2)	320 (5.6)
6	191 (3.0)	48 (8.0)	143 (2.5)
7	43 (0.7)	18 (3.0)	25 (0.4)
8	6 (0.1)	3 (0.5)	3 (0.1)
Patients with ≥ 1 topical therapy class, *n* (%)	5511 (86.6)	594 (98.3)	4917 (85.4)
Mean (SD) unique classes	2.7 (1.4)	3.8 (1.4)	2.6 (1.4)
Phototherapy use during follow‐up, *n* (%)	597 (9.4)	173 (28.6)	424 (7.4)
Time to first phototherapy, days, mean (SD)	467.4 (580.1)	512.1 (625.6)	449.1 (560.2)
Indicators to evaluate for systemic therapy eligibility			
Use of 2 topical therapies within 8 weeks, *n* (%)	4262 (67.0)	564 (93.4)	3698 (64.2)
Number of topical therapies used within first 8 weeks, mean (SD)	1.6 (0.8)	2.0 (0.9)	1.6 (0.8)
Time to initiation of third topical therapy, days, mean (SD)	438.4 (517.7)	372.3 (476.0)	451.6 (524.7)

Abbreviation: SD, standard deviation.

**FIGURE 3:**
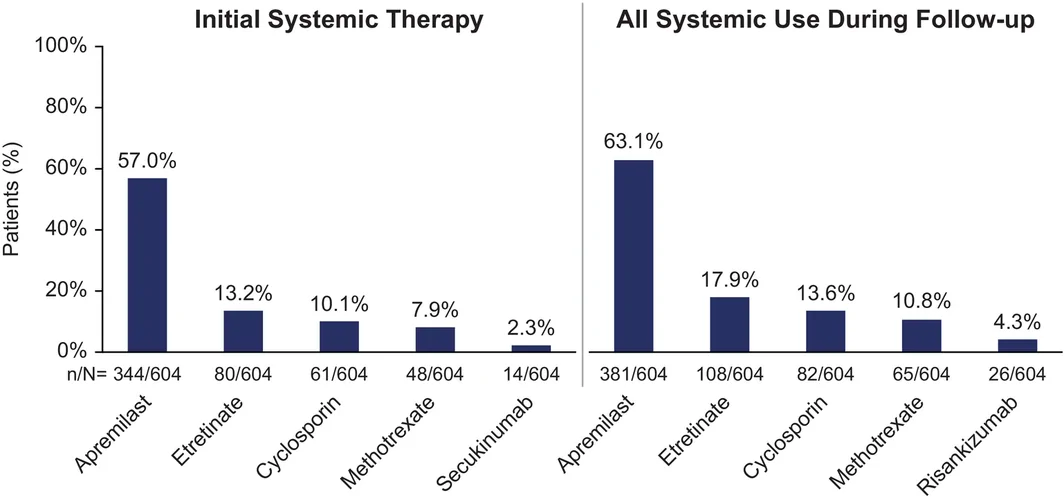
Initial systemic therapy used and all systemic therapies used during follow‐up.

The most common initial systemic therapies were apremilast (57.0%) and etretinate (13.2%; Figure 3). Compared with patients who received topical therapy alone, phototherapy was approximately 4 times more prevalent in patients who initiated systemic therapy (Table 2). Almost all (93.4%) patients who initiated systemic therapy had used 2 topical therapy classes within an 8‐week period versus 64.2% of patients who received topical therapy alone. Time to initiation of a third topical class was shorter for patients who initiated systemic therapy (372.3 days) than those who did not (451.6 days).

### 3.2 UPLIFT‐Delphi Analysis

A total of 137 UPLIFT survey respondents from Japan with PsO or PsO and PsA receiving topical therapy alone were included in the secondary analysis (Figure S4). From 10 Delphi consensus recommendations on systemic treatment eligibility, 7 corresponded with data collected in UPLIFT and were used to assess systemic treatment eligibility (Figure S2).

**FIGURE 4:**
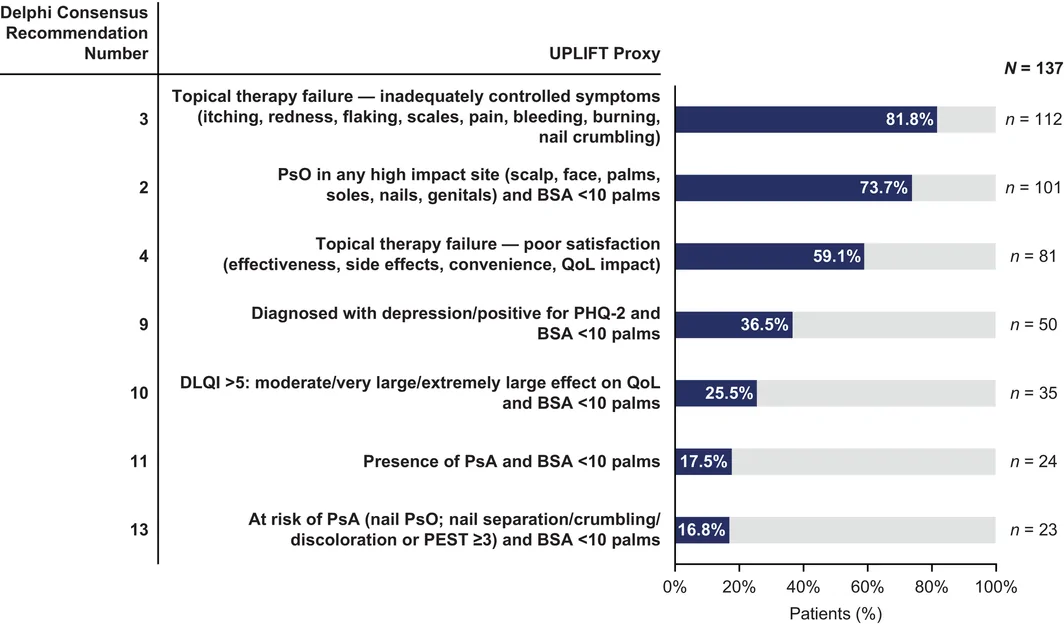
Systemic therapy eligibility in topically treated patients with PsO in UPLIFT Japan based on Delphi consensus recommendations. DLQI, Dermatology Life Quality Index; PEST, Psoriasis Epidemiology Screening Tool; PHQ‐2, Patient Health Questionnaire‐2; PsA, psoriatic arthritis; PsO, psoriasis; QoL, quality of life.

The most common indicator for systemic treatment eligibility was having an inadequate response to topical therapy, as defined by poor symptom control, observed in 81.8% of patients (Figure 4). The second most frequent indicator was PsO in high‐impact sites and BSA < 0 palms, observed in 73.7% of patients. Over half (59.1%) of patients reported low treatment satisfaction. Depression or positive PHQ‐2 scores along with BSA < 10 palms were present in 36.5% of patients; 25.5% of patients had a DLQI > 5 and BSA < 10 palms, indicating at least a moderate impact on quality of life. Presence of PsA and BSA < 10 palms was reported in 17.5% of patients, with a similar percentage (16.8%) of patients with BSA < 10 palms and at risk for PsA.

**FIGURE 5:**
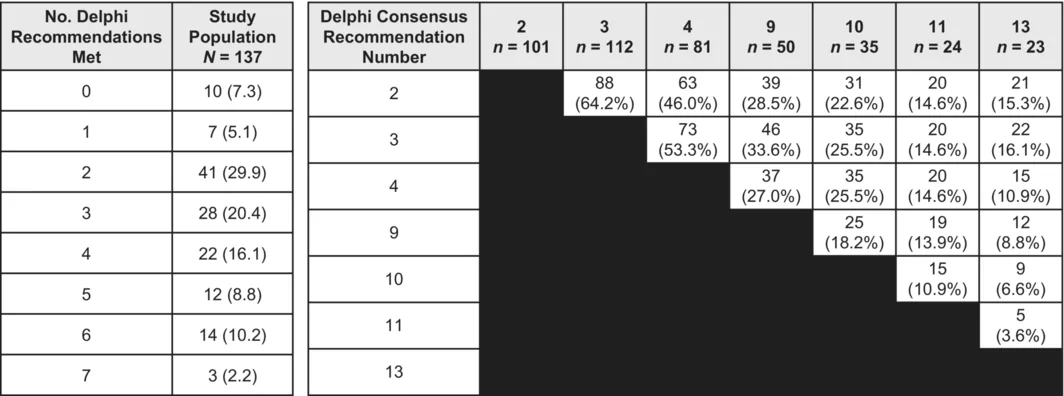
Number of patients with PsO in UPLIFT Japan who met each combination of Delphi consensus recommendations. Delphi consensus recommendation 2 UPLIFT proxy: PsO in any high‐impact site and BSA < 10 palms. Delphi consensus recommendation 3 UPLIFT proxy: Topical therapy failure—inadequately controlled symptoms. Delphi consensus recommendation 4 UPLIFT proxy: Topical therapy failure—poor satisfaction. Delphi consensus recommendation 9 UPLIFT proxy: Depression/positive PHQ‐2 and BSA < 10 palms. Delphi consensus recommendation 10 UPLIFT proxy: DLQI > 5 and BSA < 10 palms. Delphi consensus recommendation 11 UPLIFT proxy: Presence of PsA and BSA < 10 palms Delphi consensus recommendation 13 UPLIFT proxy: At risk of PsA and BSA < 10 palms.

Almost all (92.7%) patients in the UPLIFT Japan subgroup met at least 1 of the Delphi consensus recommendations; most (87.6%) met more than 1 and 57.7% met 3 or more (Figure 5). The most common combinations of consensus recommendations met were high impact site involvement with BSA < 10 palms and topical failure based on inadequate symptom control with topical therapy (64.2%) and topical failure based on inadequate symptom control and low treatment satisfaction (53.3%; Figure 5).

## 4 Discussion

This study took a 2‐pronged approach to evaluate the degree of unmet need for systemic therapy among patients with PsO in Japan by characterizing real‐world topical treatment patterns and quantifying the systemic‐eligible population. To address these aims, we used 2 complementary real‐world data sources, as no single source could both capture treatment trajectories at scale and operationalize clinically meaningful definitions of topical therapy failure. The claims analysis enabled population‐level assessment of topical cycling and longitudinal treatment pathways in routine practice, while the representative survey data from UPLIFT allowed direct mapping of a Japanese patient subgroup to Delphi consensus recommendations for systemic therapy eligibility. Together, these complementary approaches provide both breadth and interpretability, allowing characterization of topical cycling burden and application of clinically grounded recommendations for systemic therapy eligibility to estimate the size of the systemic‐eligible population in Japan.

From the retrospective analysis of the DeSC claims database, only 9.5% of patients with PsO receiving topical therapy initiated systemic therapy over a mean follow‐up of 3.8 years. Patients treated with topical therapy alone had a considerable comorbidity burden, with 28.4% having a CCI ≥ 1. Phototherapy was approximately 4 times more common among patients who initiated systemic therapy than those who received topical therapy alone. In Japan, phototherapy is often positioned between topical therapy and systemic therapy. Therefore, the higher use of phototherapy among systemic therapy initiators may reflect earlier recognition of inadequate disease control with topical therapy and a structured stepwise escalation pathway, whereby patients receiving phototherapy are more likely to be reassessed and escalated to systemic therapy when response is suboptimal. Prior to initiating systemic therapy, patients had received topical therapy alone for an average of 712 days (approximately 2 years), with 81.3% cycling through at least 3 lines of topical treatment and 11.4% cycling through at least 6 lines of topical treatment. This delay in initiating systemic treatment may lead to worse outcomes. For example, in a retrospective US cohort study, and compared with topical cycling, patients with PsO and limited skin involvement (BSA 1%–10%) experienced lower cumulative disease burden and were more likely to achieve BSA treatment goals when initiating apremilast within 6–12 months of an index BSA [19].

According to the Japanese Delphi consensus recommendations, Psoriasis Area and Severity Index > 3 or PGA ≥ 2 after 8 weeks of therapy with 2 or more topical medications, poor patient satisfaction or inadequate symptom control after 4 weeks with 2 or more topical medications constitute topical failure [12]. Similarly, the IPC recently defined topical failure as the inability to achieve clear/nearly clear skin (BSA ≤ 1% or PGA 0/1) after 2 consecutive 4‐week courses of topical therapy [13]. Thus, despite most patients in the DeSC database remaining on topical therapy alone, the observed lack of systemic escalation may not necessarily indicate topical success, and many patients may meet criteria for topical failure without treatment escalation. Among patients in the DeSC database treated with topical therapy alone, 64.2% used 2 topical therapies within 8 weeks, suggesting these patients should be evaluated for topical treatment failure at week 8.

It is important to note that 75.7% of all patients in our DeSC claims data analysis disenrolled, died, or discontinued topical therapy during follow‐up. It is possible some individuals improved clinically, re‐initiated topical therapy after the end of the study period, or were lost to follow‐up due to changes in insurance or other reasons that were not captured, an inherent limitation of this database.

Almost all (92.7%) Japanese patients from UPLIFT receiving topical therapy alone met at least 1 Delphi recommendation for systemic treatment eligibility. The most common indicator for systemic eligibility was topical therapy failure, with 81.8% of patients experiencing poor symptom control and 59.1% reporting low treatment satisfaction with topical therapy alone. Additionally, 73.7% of patients had lesions in high‐impact sites. These findings indicate that, based on real‐world disease burden and patient‐reported outcomes, a substantial number of patients currently on topical therapy alone may be eligible for systemic treatment. This aligns with a previous retrospective cohort study of the Optum deidentified Electronic Health Records database in the US, which found that of 1733 patients treated with topical therapy only, 28.7% had BSA > 10% and 48.3% had PsO in 2 or more high‐impact sites, indicating that many patients who may be eligible for systemic therapy remain on topical therapy alone [21, 23]. Gaps in communication between patients and physicians, and a disconnect between patient and physician perceptions of PsO disease severity and treatment outcomes, may contribute to this undertreatment [15]. The previous lack of a clear definition of topical failure until the recent Japanese Delphi consensus study and IPC guidelines may also be a factor contributing to undertreatment. Increasing awareness of IPC guidelines, implementing Delphi‐based treatment recommendations in clinical practice, and improving communication between patients and physicians could help reduce undertreatment.

This study has several limitations. The primary analysis was based on claims data, which may not be accurate or complete. For example, patients may not use medications as prescribed or medical providers may not record certain diagnoses. Additionally, when days supplied were not recorded, assumptions were made regarding the length of treatment with topical and systemic therapies. Claims data do not provide key clinical information such as disease severity, symptoms, and quality of life. Therefore, the DeSC claims analysis was not intended to directly measure clinical treatment response or define topical treatment failure, but rather to descriptively characterize topical treatment patterns, topical cycling, and systemic therapy initiation. Treatment pattern variables, such as number of topical therapy classes used and use of ≥ 2 topical therapies within 8 weeks, were interpreted as milestones where clinical reassessment for potential systemic therapy eligibility may be relevant, not as definitive measures of treatment failure. Systemic initiation was evaluated as an observed treatment‐pattern outcome during follow‐up. Because claims data do not capture disease severity, symptoms, quality of life, physician rationale, or patient preference, systemic therapy initiation should not be interpreted as a definitive indicator of topical treatment failure or insufficient clinical response. There may also be selection bias in the DeSC patient population; the mean age of patients included for analysis was 66.8 years and 58.3% were men, which is older than the overall PsO population in Japan and a lower proportion of men according to epidemiological analysis (mean age: 53.8 years, 65.3% men) [16]. For the secondary analysis, the UPLIFT survey data are based largely on self‐reported patient information, which has inherent limitations, including poor recall of prior health status or diagnoses. For some of the Japanese Delphi recommendations, there were no relevant proxies in UPLIFT. Furthermore, where proxies did exist, they are likely an imperfect measure of the corresponding consensus recommendation and many were subjective in nature. The validity of these proxies has also not been clearly established and should be further evaluated in future studies. Both analyses were limited to patients with no prior systemic treatment, which may have resulted in a bias toward patients with milder disease and should be considered when interpreting findings related to potential undertreatment.

Taken together, these findings suggest a potential gap in the management of PsO in Japan. Despite prolonged and repeated use of topical therapies, many patients experience inadequate disease control and impaired quality of life—conditions that meet recently‐established criteria for systemic therapy. Greater alignment between clinical practice and evolving treatment recommendations, including earlier recognition of topical treatment failure and timely escalation to systemic treatment, could meaningfully improve long‐term patient outcomes and reduce the physical and psychosocial burden of PsO.

## Funding

This work was supported by Amgen Inc. Thousand Oaks, California, USA.

## Ethics Statement

The authors have nothing to report.

## Conflicts of Interest

A.M. has received research grants, consultancy fees, and/or speaker's fees from AbbVie, Amgen, Boehringer Ingelheim, Bristol Myers Squibb, Eli Lilly Japan, Johnson&Johnson, Kyowa Kirin, LEO Pharma, Maruho, Sun Pharma Japan, Taiho Pharmaceutical, Torii Pharmaceutical, UCB Japan, and Ushio. S.I. is a member on the Editorial Board of *Journal of Dermatology* and author of this article. To minimize bias, they were excluded from all editorial decision‐making related to the acceptance of this article for publication. S.I. has also received grants and/or personal fees from AbbVie, Amgen, Bristol Myers Squibb, Daiichi Sankyo, Eisai, Eli Lilly, Janssen Pharma, Kyowa Kirin, LEO Pharma, Maruho, Novartis, Sun Pharma, Taiho Yakuhin, Torii Yakuhin, and UCB. H.T. has received speaker's fees from AbbVie, Amgen, Bristol Myers Squibb, Eli Lilly, Janssen Pharma, Kyowa Kirin, LEO Pharma, Maruho, Novartis, Sun Pharma, Taiho Pharmaceutical, and UCB Japan. L.P. and S.C. are employees and shareholders of Amgen Inc. H.M. and J.L. are employees and shareholders of Amgen K.K.

## Supporting information


**Figure S1:** Study design schema.
**Figure S2:** Japanese Delphi consensus recommendations included for analysis and corresponding data proxies in Japan subgroup of UPLIFT Survey.
**Figure S3:** Patient disposition for DeSC claims analysis.
**Figure S4:** Patient disposition for UPLIFT‐Delphi analysis.

## Data Availability

The datasets generated during and/or analyzed during the current study may be requested at http://www.amgen.com/datasharing.
