# Supplementary material for: Topical Cycling and Systemic Eligibility in Patients With Psoriasis in Japan
Source: J Dermatol. 2026 Jul 24;53(9):1277–86. doi: 10.1111/1346-8138.70395 (PMC13555188; doi:10.1111/1346-8138.70395)
Supplement: Supplementary file 1 [file JDE-53-1277-s001.docx]

**Supplementary Tables and Figures**

**Supplementary Figure S1** Study design schema

**A. DeSC Claims Analysis**

**B. UPLIFT-Delphi Analysis**

DLQI, Dermatology Life Quality Index; EHI, Employee Health Insurance; LSEHS, Latter-Stage Elderly Healthcare System; NHI, National Health Insurance; PEST, Psoriasis Epidemiology Screening Tool; PHQ-2, Patient Health Questionnaire-2; PsA, psoriatic arthritis; PsO, psoriasis; UPLIFT, Understanding Psoriatic Disease Leveraging Insights for Treatment.

**Supplementary Figure S2** Japanese Delphi consensus recommendations included for analysis and corresponding data proxies in Japan subgroup of UPLIFT Survey

 BSA, body surface area; DLQI, Dermatology Life Quality Index; PEST, Psoriasis Epidemiology Screening Tool; PHQ-2, Patient Health Questionnaire-2; PsA, psoriatic arthritis; PsO, psoriasis.

**Supplementary Figure S3** Patient disposition for DeSC claims analysis

PsO, psoriasis.

**Supplementary Figure S4** Patient disposition for UPLIFT-Delphi analysis

PsO, psoriasis; PsA, psoriatic arthritis.
